# METTL13 inhibits progression of clear cell renal cell carcinoma with repression on PI3K/AKT/mTOR/HIF-1α pathway and c-Myc expression

**DOI:** 10.1186/s12967-021-02879-2

**Published:** 2021-05-13

**Authors:** Zhuonan Liu, Tianshui Sun, Chiyuan Piao, Zhe Zhang, Chuize Kong

**Affiliations:** 1 Department of Urology, First Hospital of China Medical University, School of China Medical University, No. 155 Nanjing North Street, Heping District, Shenyang City, 110004 Liaoning Province People’s Republic of China; 2 Department of Obstetrics and Gynecology, Shengjing Hospital of China Medical University, Shenyang City, 110004 Liaoning Province People’s Republic of China

**Keywords:** METTL13, Clear cell renal cell carcinoma, Proliferation, Migration, Invasion, Epithelial-mesenchymal transition, HIF-1α, C-Myc

## Abstract

**Background:**

Clear cell renal cell carcinoma (ccRCC) is the most common and aggressive type of renal malignancy. Methyltransferase like 13 (METTL13) functions as an oncogene in most of human cancers, but its function and mechanism in ccRCC remains unreported.

**Methods:**

qRT-PCR, western blotting and immunohistochemistry were used to detect METTL13’s expression in tissues. The effects of METTL13 on ccRCC cells’ growth and metastasis were determined by both functional experiments and animal experiments. Weighted gene co-expression network analysis (WGCNA) was performed to annotate METTL13’s functions and co-immunoprecipitation (co-IP) was used to determine the interaction between METTL13 and c-Myc.

**Results:**

METTL13 was underexpressed in ccRCC tissues compared to normal kidney tissues and its low expression predicted poor prognosis for ccRCC patients. The *in vitro* studies showed that knockdown and overexpression of METTL13 respectively led to increase and decrease in ccRCC cells’ proliferation, viability, migratory ability and invasiveness as well as epithelial-mesenchymal transition (EMT). The *in vivo* experiment demonstrated the inhibitory effect that METTL13 had on ccRCC cells’ growth and metastasis. Bioinformatic analyses showed various biological functions and pathways METTL13 was involved in. In ccRCC cells, we observed that METTL13 could negatively regulate PI3K/AKT/mTOR/HIF-1α pathway and that it combined to c-Myc and inhibited c-Myc protein expression.

**Conclusions:**

In general, our finding suggests that high expression of METTL13 is associated with favorable prognosis of ccRCC patients. Meanwhile, METTL13 can inhibit growth and metastasis of ccRCC cells with participation in multiple potential molecular mechanisms. Therefore, we suggest METTL13 can be a new diagnostic and therapeutic target for ccRCC in the future.

**Supplementary Information:**

The online version contains supplementary material available at 10.1186/s12967-021-02879-2.

## Background

As the most frequently diagnosed cancer of kidney and the ninth most common of all types of cancer, renal cell carcinoma (RCC) was estimated to cause 14,770 deaths in United States with 73,820 new cases reported in 2019 [1] and the situation in China was similar [2]. Resection is still the major and the most effective therapeutic method for localized RCC, while metastasis occurs in approximate 25% cases, which makes it difficult for patients to undergo surgery [3]. Among all the histological and molecular subtypes of RCC, clear cell RCC (ccRCC) is the most common subtype, accounting for about 75% [1, 4]. Besides, ccRCC is resistant to radiotherapy and chemotherapy, which makes patients’ prognosis far from satisfying [5, 6]. Thus, it’s urgent and important to identify crucial biomarkers for ccRCC and to have comprehensive insights into deep molecular mechanisms playing in this neoplasm with the aim to provide molecular bases and inspirations for the diagnosis, monitoring and treatment.

Protein methyltransferase like 13 (METTL13), also called FEAT, is coded by gene METTL13, which is located at chromosome 1q24.3. Purified from rat livers in 2011 by a Japanese researcher, METTL13 was found to be abnormally overexpressed in several human cancers including lung cancer and to drive tumorigenesis in transgenic mice [7–10]. A study indicated that METTL13 inhibits apoptosis of lung, breast and liver cancer cells and miR-16 can repress the expression of METTL13 by binding to its 3′-untranslated region [8]. In 2016, researchers detected high expression levels of METTL13 in the plasma of patients with breast, ovarian and lung cancer [9], following which it was identified as a recurrence predictor for breast cancer [10]. As a methyltransferase protein, it can specifically methylate the Lys55 of eEF1A, resulting in the increase of its translational output and tumorigenesis of lung and pancreatic cancer [11, 12]. In 2019, scholars demonstrated that expression of METTL13 is positively regulated at transcriptional level by HN1L and METTL13 can enhance hepatocellular carcinoma development by up-regulating TCF3 and ZEB1 [13]. Despite the studies above proving the oncogenicity role of METTL13, an article elucidated its downregulated expression in bladder cancer and its tumor-suppressing functions [14]. However, studies targeted at METTL13 are still very scarce and unintegrated with uncertainty of its roles in various cancers; meanwhile, its expression and biological functions in ccRCC remain unknown, which are worth further exploration.

In this study, it was revealed that METTL13 was underexpressed in ccRCC tissues compared to normal kidney and its low expression was associated with unfavorable prognosis. METTL13 was detected by us to have significant inhibitory effect on growth and metastasis of ccRCC cells. Besides, METTL13 could repress PI3K/AKT/mTOR/HIF-1α signaling pathway as well as c-Myc expression and might participate in other potential mechanisms, including metabolism regulation and cell adhesion alteration. These findings may provide insights into better understanding of METTL13’s molecular functions in ccRCC as well as inspiration for ccRCC diagnosis and therapy.

## Materials and methods

### Bioinformatic analyses

Website UALCAN (http://ualcan.path.uab.edu/) was used to obtain gene expressions in different sample types (533 ccRCC tissues and 72 normal kidney tissues), different pathological grades and clinical stages of ccRCC with transcriptome data provided by The Cancer Genome Atlas (TCGA) database. METTL13 expressions in 72 pairs of ccRCC tissues and adjacent normal tissues were also acquired from a dataset (GSE53757) of the Gene Expression Omnibus (GEO) database (http://www.ncbi.nlm.nih.gov/geo/) [15]. Website GEPIA (http://gepia.cancer-pku.cn/) directly produced survival curves of ccRCC patients with high and low METTL13 expression levels based on an appropriate expression threshold. Transcriptome data of kidney renal clear cell carcinoma (KIRC) cohort was downloaded from TCGA database and differentially expressed genes (DEGs) was filtered out with |logFC|> 1 and false discovery rate (FDR) < 0.05 as the criteria. We performed weighted gene co-expression network analysis (WGCNA) with the WGCNA package in R (The WGCNA package in R). ClusterProfiler R package was used to determine gene modules’ gene ontology and KEGG pathway enrichments regarding FDR < 0.05 as threshold.

### Patient samples

All the ccRCC tissues and their corresponding adjacent normal tissues were obtained from the urology surgery department of the first hospital of China Medical University (Shenyang, China). 50 pairs of ccRCC tissues and corresponding adjacent normal kidney tissues were respectively analyzed by qRT-PCR and 13 pairs were analyzed by western blotting assay. 48 ccRCC tissues were used for immunohistochemistry analysis. This study was approved by Research Ethics Committee of China Medical University (No: AF-SOP-07-1. 1-01, Additional file 1) and all the patients had supplied the written informed consent.

### Cell lines and cell culture

Human cell lines, 786-O, 769-P, OS-RC-2, Caki-1 and ACHN were purchased from Chinese Academy of Sciences Type Culture Collection Cell Bank (Shanghai, China). 786-O, 769-P and OS-RC-2 cells were cultured in RPMI medium (Hyclone; GE Healthcare), while Caki-1 cells and ACHN cells were treated with McCoy's 5A medium Hyclone; GE Healthcare) and MEM medium (Hyclone; GE Healthcare), respectively. All the mediums contained 10% fetal bovine serum (FBS; Biological Industries, Beit-HaEmek, Israel) and cells were cultured at 37°C with 5% CO2.

### Cell transfection

Two strands of small interfering RNA (siRNA) targeting at METTL13 were designed and purchased from JTSBIO Co. (China), sequences of which were as following: si-METTL13#1 (sense: GCGGGGUGCUACAUAAAUATT; anti-sense: UAUUUAUGUAGCACCCCGCTT), si-METTL13#2 (sense: GCUCUGCCCUUCAGAUCUUTT; anti-sense: AAGAUCUGAAGGGCAGAGCTT). Usage of LipofectamineTM3000 (Invitrogen, USA) was involved in siRNA transfection with the protocol provided by manufacturer's guidelines. METTL13 overexpression plasmid was purchased from GeneChem (Shanghai, China) and transfection was performed according to manufacturer's instructions.

### Quantitative real-time PCR (qRT-PCR)

Total RNA was extracted from tissue samples and cells with the use of RNAiso Plus (Takara Biotechnology, Dalian, China) according to manufacturer’s instructions and subsequently the Prime Script RT Master Mix (Takara Biotechnology, Dalian, China) was utilized to conduct reverse transcription to synthesize cDNA. qRT-PCR was performed by Sybr Premix Ex Taq TMKit (Takara Biotechnology, Dalian, China) and LightCyclerTM 480 II system (Roche, Basel, Switzerland), after which the 2^−ΔΔCt^ method was used to calculate the relative expression level of each sample referring to internal β‐actin or GAPDH expression. Information of primer sequences is listed in Additional file 2: Table S1.

### Western blotting assay

Total protein from patient samples or cells was obtained by RIPA lysis buffer with 1% Phenylmethylsulfonyl fluoride (PMSF) contained and protein concentrations were detected by bicinchoninic acid (BCA) assay kit (Beyotime Institute of Biotechnology). Equal mass of proteins (30 μg/lane) were used for electrophoresis in 10% SDS-polyacrylamide, followed by PVDF membrane (0.2 μm) transfer, membrane blocking by 5% non-fat milk, primary and secondary antibody incubation as well as image capture by an EasySee Western Blot kit (Beijing Transgen Biotech, Beijing, China) and a chemiluminescence system (Bio-Rad, CA, USA). Information of primary antibodies is listed in Additional file 3: Table S2.

### 5-Ethynyl-2′-deoxyuridine (EdU) assay

EdU assay was performed with the usage of an EdU kit (BeyoClickTM, EDU-488, China). Cells were co-cultured with EdU working solution (1:1000) at 37 °C in a humidified 5% CO2 atmosphere for 2–4 h, followed by fixation with 4% paraformaldehyde for 30 min and treatment with 0.3% Triton X-100 for 30 min at room temperature. Then, according to the manufacture’s protocol, cells were co-incubated with click reaction solution for 30 min at room temperature in a dark environment, after which cells were treated with Hoechst solution for 10 min. We used a fluorescence microscope (Olympus Corporation, Japan) to capture images with a magnification of 200× and cell counting was conducted by ImageJ software.

### Cell viability assay (CCK-8 assay)

Counting Kit-8 (CCK-8) solution (Bimake, USA) was added to each well of 96-well plates to the concentration of 0.5 mg/ml, where 2.0 × 10^3^ cells had been initially loaded. After incubation for one hour at 37 °C with 5% CO_2_, absorbance at 450 nm was measured by plate reader (Model 680; Bio-Rad Laboratories).

### Wound-healing assay

When the density of cells in 6-well plates reached above 90%, we used a 1-mL pipette tip to vertically scratch an artificial wound in the middle of the wells. Then cells were washed with PBS and new FBS-free medium was added. Images were obtained with the help of an inverted microscope (EVOS XL system, AMEX1200; Life Technologies Corp, Bothell, WA, USA) at 40 X magnification. After cultured for 48 h, cell images were re-obtained.

### Cell migration and invasion assay

8-μm-pore transwell chambers in 24-well plates (Corning Costar, Corning, NY, USA) were used. Chambers coated with Matrigel (BD, San Diego, CA, USA) were for cell invasion detection, while those without Matrigel coating were used to determine cell migration. 600 μl 10%-FBS containing medium was placed into each bottom chamber, while equal number of suspended cells (1.0–1.5 × 10^4^ cells for migration assay, 3.0–4.0 × 10^4^ cells for invasion assay) in 200 μl medium without FBS were imbedded onto each upper chamber. After cultured at 37 °C with 5% CO2 for 48 h, suspended cells in the upper chamber were washed out, while cells adhering to the bottom membrane were stained by crystal violet. Images were obtained by using the inverted microscope described previously at 100X magnification and cell counting was performed by software *Image J*.

### Co-immunoprecipitation (Co-IP) assay

Cells were lysed by RIPA lysis buffer containing 1% PMSF and 1% protease inhibitor. A certain amount of cell lysate was isolated as input, while 5 μg primary antibody (METTL13, abcam, ab186002; c-Myc, Santa Cruz, sc-40) or homologous IgG (Santa Cruz Biotechnology) was co-incubated with remaining lysate at 4 °C overnight. Then, 30-μl protein A/G-beads was co-incubated with the lysis solution at 4 °C for an hour, after which beads was extracted and washed by washing buffer three times. Next, proteins were isolated by using beads into 2x protein loading buffer after co-incubation at 100 °C for 15 min and western blot was finally conducted.

### Immunohistochemistry assay

Tissues previously formalin-fixed and paraffin-embedded were cut into 4-μm slices and they were treated according to procedures previously described [16], which involved use of rabbit anti-METTL13 antibody (GTX120626, GeneTex, USA) and an UltraSensitiveTM SP (Mouse/Rabbit) IHC kit (Maxin-Bio, Fuzhou, Fujian, China) according to the manufacture’s guidance. Images were captured by the inverted microscope with magnifications of 200× and 400×.

### Animal experiments

Experiments with animals involved were approved by China Medical University Ethics Committee of Medical Experimental Animal Welfare and were conducted following the institute’s guidelines. 14 female BALB/c-nude mice of 4–6 weeks old, purchased from Beijing Vital River Experimental Animal Technology Co. Ltd., were housed in a pathogen-free environment at Experimental Animal Department of China Medical University. As for the tumorigenicity study, 1.0 × 10^6^ OS-RC-2 cells (empty vector or METTL13 overexpression) in 150 μl serum-free 1640 medium containing 40% Matrigel were injected subcutaneously into flank of each mouse, 30 days after which mice were euthanized and tumors were excised. Each group included 4 mice. Primary tumors were measured for their size and weight. As for the metastasis study, 1.0 × 10^5^ OS-RC-2 cells (empty vector or METTL13 overexpression) in 150 μl pathogen-free PBS were injected into per mouse via its lateral tail vein. After 45 days, lungs were separated and metastatic tumors were counted. Each group included 3 mice. Hematoxylin and eosin staining was applied in observing serial histological sections of the lungs.

### Statistical analysis

Each experiment was performed independently for at least 3 times and data were expressed as the mean ± standard deviation (SD). Software GraphPad Prism of version 8.0 (La Jolla, CA, USA) was used to perform all the statistical analysis. Differences between two groups were evaluated by Student's t test. Differences in expression for samples with paired measures were analyzed by Wilcoxon signed rank test. Survival status was analysed by Kaplan‐Meier/Logrank methods. As for all the data, P < 0.05 was regarded as statistically significant. * indicates P < 0.05; ** indicates P < 0.01, *** indicates P < 0.001.

## Results

### Low expression of METTL13 is associated with poor outcome of ccRCC

**Fig. 1:**
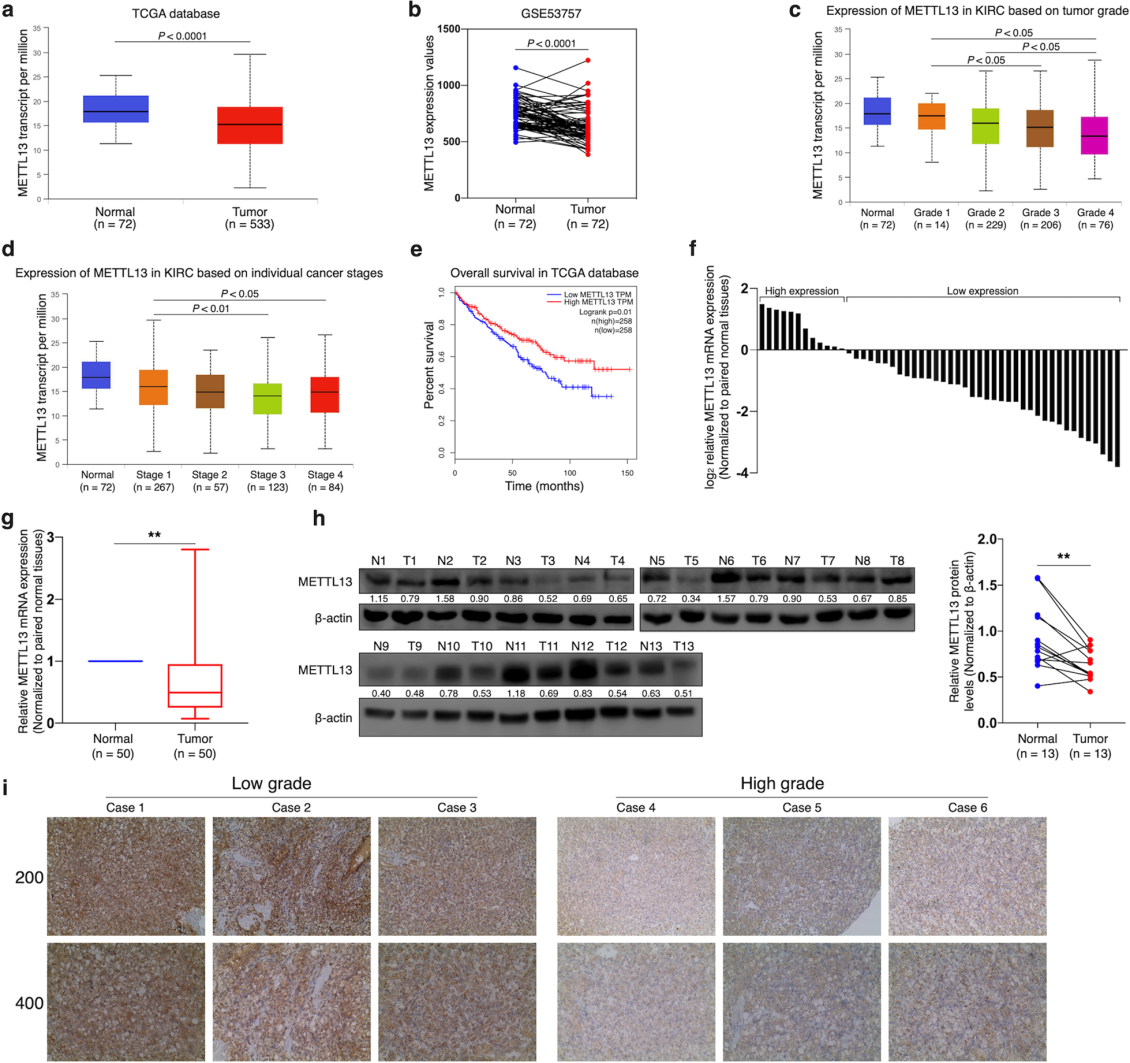
Low expression level of METTL13 is associated with poor prognosis of ccRCCs. **a**, **b** METTL13 expression levels in ccRCC tissues and normal kidney tissues according to TCGA database (**a**) and GSE53757 dataset (**b**). **c**, **d** Expression levels of METTL13 in ccRCC tissues of different tumor grades (**c**) and of different cancer stages (**d**) in TCGA dataset. **e** Kaplan–Meier analysis of ccRCC patients’ overall survival based on METTL13 expression in TCGA database. **f**, **g** Relative mRNA levels of METTL13 in 50 ccRCC tissues and their corresponding adjacent normal kidney tissues detected by qRT-PCR assay. **h** METTL13 protein levels in 13 pairs of ccRCC tumors (T) and their corresponding adjacent normal kidney tissues (N) tested by western blotting. **i** IHC staining of METTL13 in ccRCC tissues of different pathological grades. Mann-Whitney U-test was performed for statistical analysis for qRT-PCR and western blotting results. **P < 0.01

Despite the oncogenic role of METTL13 in most of human cancers, not only TCGA database but also GEO dataset showed that METTL13 was underexpressed at transcriptional level in ccRCC tissues (Fig. 1a, b). In addition, by using the website UALCAN, we detected that METTL13 expression levels were significantly and negatively correlated to tumor grades and to cancer stages of ccRCC (Fig. 1c, d). According to Kaplan-Meier survival curves provided by GEPIA on the basis of TCGA database (Fig. 1e), it was found that ccRCC patients with higher METTL13 expression levels were more likely to have better prognosis (P = 0.01). By analyzing 50 pairs of samples using qRT-PCR assay, we observed significant decrease of METTL13 mRNA expression in ccRCC tissues compared to that in adjacent normal tissues (Fig. 1f, g). The result of western blotting also indicated the underexpression of METTL13 in ccRCC tissues at protein level (Fig. 1h). Immunohistochemistry results suggested that METTL13 protein expression declined with increase of tumor grade (Fig. 1i). Therefore, we could suggest that low expression of METTL13 was significantly associated with ccRCC occurrence and unfavorable prognosis of ccRCC patients.

### Knockdown of METTL13 promotes ccRCC cells’ proliferation, migration and invasion

**Fig. 2:**
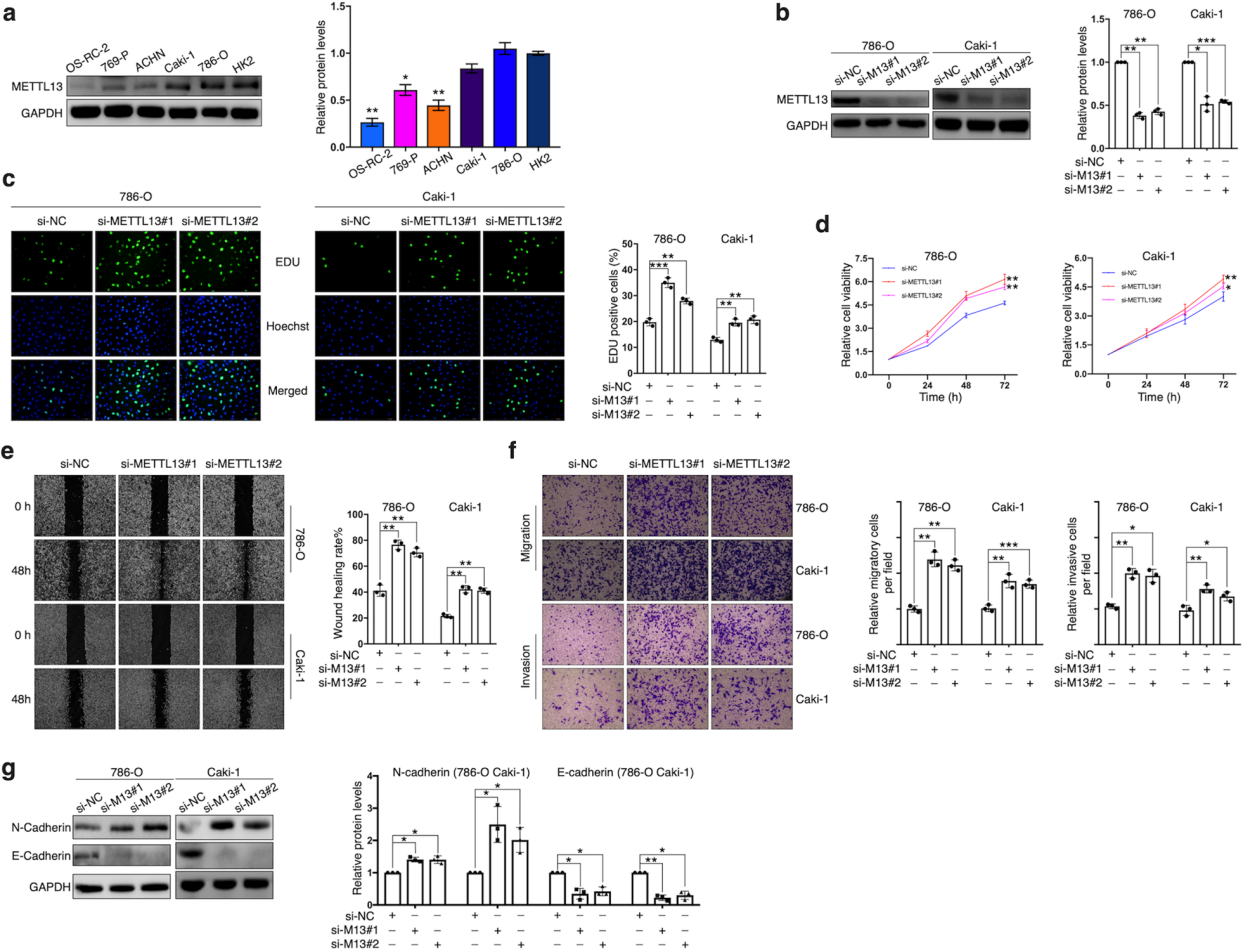
Knockdown of METTL13 promotes ccRCC cell proliferation, viability, migration and invasion. **a** METTL13 protein expressions in five ccRCC cell lines and one immortalized ccRCC cell line by western blotting. **b** Knockdown efficiencies of two siRNAs targeting METTL13 in two cell lines detected by western blotting. **c-e** Cell proliferation, viability and migration were measured by EdU assay (**c**), CCK-8 assay (**d**) and wound-healing assay (**e**), respectively. **f** Cell migration assay and invasion assay were respectively used to detect cells’ migratory ability and invasiveness. **g** Expression levels of EMT-related proteins (N-cadherin, E-cadherin) from cells were detected via western blotting. Each experiment was conducted independently at least three times. Student’s t-test (two-tailed) was used for statistical analysis, *P < 0.05, **P < 0.01, ***P < 0.001. Bar graphs: mean ± S.D., n = 3

Results of METTL13 protein expression obtained from 5 ccRCC cell lines (OS-RC-2, 760-P, ACHN, Caki-1 and 786-O) and a normal renal proximal tubule epithelial cell line (HK-2) showed that METTL13 was significantly underexpressed in most of the cancer cell lines (Fig. 2a). According to the result, two cell lines, 786-O and Caki-1, in which METTL13 was expressed relatively high, were selected to perform functional experiments with by knocking down METTL13 expression. Two strands of siRNA targeted at METTL13 were designed and their knockdown efficiencies were validated by immunoblotting assay (Fig. 2b). After transfecting the siRNA, we found that proliferation and viability of the two cell lines were significantly enhanced (Fig. 2c, d). In addition, knockdown of METTL13 respectively led to increase in wound healing rates of 786-O cells and Caki-1 cells (Fig. 2e), demonstrating its promotion of cell migration, furtherly validated by migration assay (Fig. 2f). Meanwhile, silencing METTL13 expression significantly improved cells’ invasiveness (Fig. 2f). Western blotting results revealed that N-cadherin expression was upregulated in METTL13-silenced ccRCC cells, whereas E-cadherin was distinctly decreased (Fig. 2g). In general, inhibition of METTL13 expression facilitated proliferation, viability, migration and invasion of ccRCC cells as well as epithelial-mesenchymal transition.

### METTL13 inhibits ccRCC cells’ proliferation, migration and invasion

**Fig. 3:**
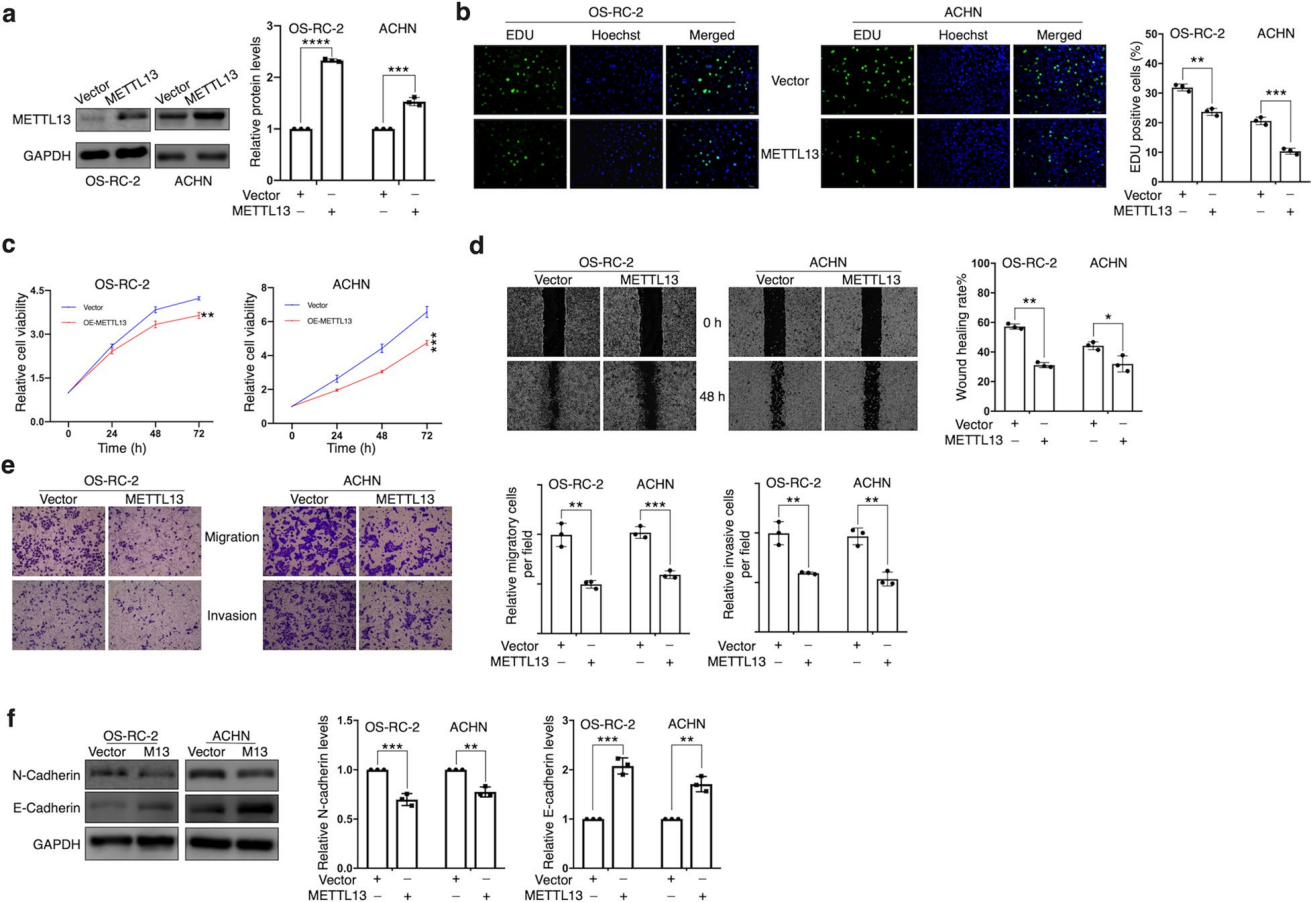
Overexpression of METTL13 inhibits proliferation, viability, migration and invasion of ccRCC cells *in vitro*. **a** METTL13 protein levels in ccRCC cells (OS-RC-2 and ACHN cells) respectively transfected with METTL13 expressing plasmid (METTL13) and control vector (vector) confirmed by western blotting. **b** Cell proliferation was detected by EdU assay. **c** CCK-8 assay was performed to measure cell viability. **d** Cell migration rate was observed via wound-healing assay. **e** Migratory ability and invasiveness of ccRCC cells were detected by migration assay and invasion assay, respectively. **f** EMT-related protein levels were detected by performing western blotting. Each experiment was conducted independently triplicate. Student’s t-test (two-tailed) was used for statistical analysis, *P < 0.05, **P < 0.01, ***P < 0.001. Bar graphs: mean ± S.D., n = 3

Then by using lentiviral vector expressing METTL13, we respectively constructed METTL13 stable-overexpressed OS-RC-2 and ACHN cell lines (Fig. 3a). Results of EdU assay suggested significant decrease in proliferating cells’ portion after overexpressing METTL13 expression (Fig. 3b). In accordance with our expectation, METTL13 overexpression gave rise to not only cell viability inhibition (Fig. 3c) but also to restraint on cell migration and invasiveness (Fig. 3d, e). After overexpressing METTL13, the alterations in expressions of EMT-related proteins, N-cadherin and E-cadherin, were shown in (Fig. 3f), which were opposite to what resulted from siRNA disposals, indicating METTL13’s role in inhibiting EMT of ccRCC cells. These data suggested upregulation of METTL13 inhibited growth, metastasis and EMT of ccRCC cells.

### Functional correlations of METTL13

**Fig. 4:**
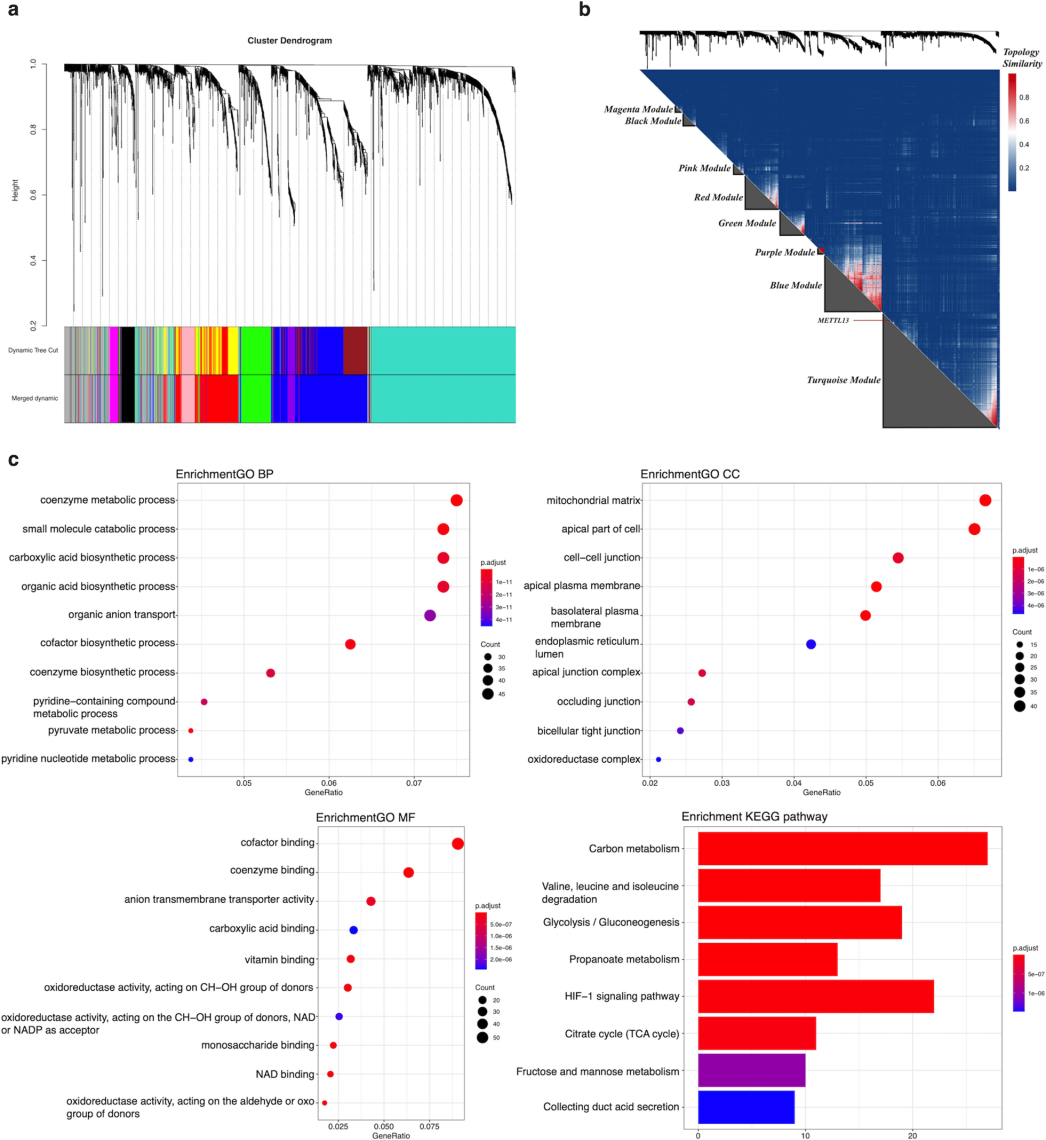
Functional annotation of METTL13 in ccRCC. **a** DEGs were divided into modules by weighted gene co-expression network analysis (WGCNA). **b** Correlation analysis of genes and identification of METTL13 location in modules. **c** GO and KEGG functional enrichment analyses of the turquoise module. GO, gene ontology; KEGG, Kyoto Encyclopaedia of Genes and Genomes; BP: biology process; CC: cellular component; MF: molecular function

**Fig. 5:**
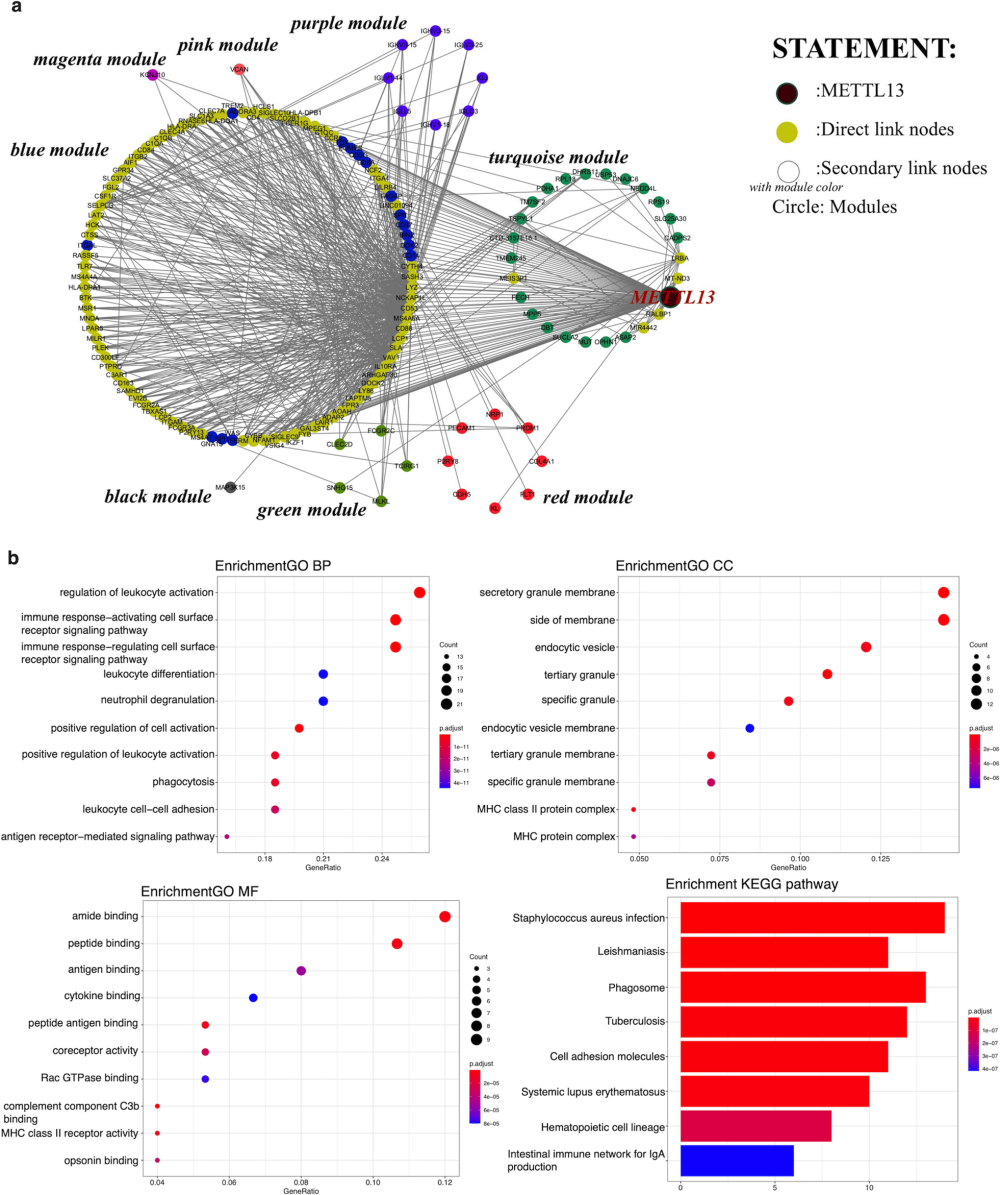
Functional annotation of genes directly linked to METTL13 in WGCNA. **a** Network of nodes in METTL13’s secondary connection. **b** GO and KEGG functional enrichment analyses of genes directly linked to METTL13 in WGCNA. GO, gene ontology; KEGG, Kyoto Encyclopaedia of Genes and Genomes; BP: biology process; CC: cellular component; MF: molecular function

The next step to investigate METTL13’s potential molecular mechanisms in ccRCC was performed by bioinformatic analyses with data obtained from TCGA database. After standardization, we inserted all the differently expression genes (DEGs) in ccRCC including METTL13 into WGCNA. The merged dynamic result showed that genes were divided into eight modules, the black, blue, green, magenta, pink, purple, red and turquoise ones (Fig. 4a). METTL13 and genes with similar expression mode were located in the turquoise module (Fig. 4b). Functional enrichment analysis of the turquoise module shown in Fig. 4c suggested that METTL13 had tremendous potential to participate in metabolism regulations, including metabolism-related pathways like the HIF-1 signaling pathway. Furthermore, by extracting nodes in METTL13’s secondary connection and drawing network, we noticed that despite location in turquoise module, METTL13 directly connected to many genes situated in the blue module, which share high correlation with METTL13 (Fig. 5a). Meanwhile, the interactions between METTL13 and other modules were mainly dependent on the blue module. As for genes directly linked to METTL13, results of functional enrichment analyses were shown in Fig. 5b, based on which we predict that METTL13 affects tumor metastasis not only via EMT regulation but also by modulating cell adhesion. Taken together, METTL13 might regulate various biological functions as well as signaling pathways in ccRCC.

### METTL13 inhibits PI3K/AKT/mTOR/ HIF-1α signaling pathway in ccRCC

**Fig. 6:**
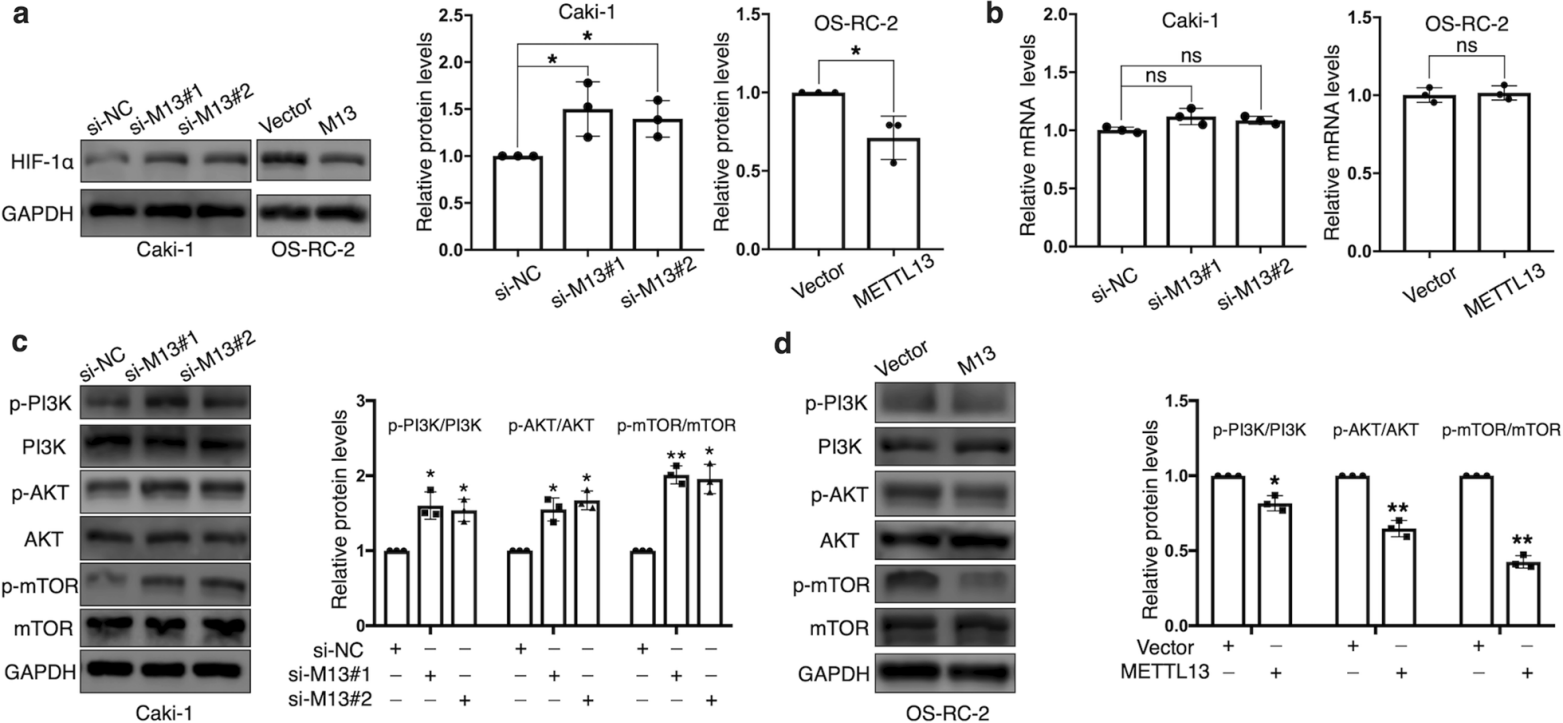
METTL13 negatively regulates PI3K/AKT/mTOR/HIF-1α signaling pathway. **a**, **b** Protein levels (**a**) and mRNA levels (**b**) of HIF-1α were respectively detected by western blot and qRT-PCR after silencing or overexpressing METTL13. **c, d** Protein levels of p-PI3K, PI3K, p-AKT, AKT, p-mTOR and mTOR were determined by western blot after knocking down METTL13 (**c**) or overexpressing METTL13 (**d**). Each experiment was carried out at least for three times independently. Student’s t-test (two-tailed) was used for statistical analysis, *P < 0.05, **P < 0.01, ***P < 0.001. Bar graphs: mean ± S.D., n = 3

With the guidance of bioinformatic analyses, we noticed that METTL13 participated in regulation of HIF-1 signaling pathway. As a core factor participating in HIF-1 signaling pathway, hypoxia-inducible factor-1α (HIF-1α) has been reported to affect multiple biological behaviors of renal cell carcinoma cells [17, 18]. Then, we tried to figure out the impact that METTL13 had on HIF-1α. Results showed that silencing METTL13 resulted in significantly increase in HIF-1α protein levels in Caki-1 cells and on the contrary in OS-RC-2 cells, the overexpression of METTL13 led to an opposite effect (Fig. 6a). However, HIF-1α mRNA expressions were not influenced by METTL13 expression alterations (Fig. 6b), which suggested METTL13 might regulate HIF-1α in a post-transcriptional manner. It’s known that PI3K/AKT/mTOR signaling pathway importantly participates in HIF-1α protein translation [17] and after we respectively silenced and overexpressed METTL13, we detected that METTL13 could negatively regulate the phosphorylation levels of PI3K, AKT and mTOR without obvious impact on the total protein expressions (Fig. 6c, d). Taken together, we suggested that METTL13 could inactivate the PI3K/AKT/mTOR/HIF-1α pathway in ccRCC cells.

### METTL13 binds to c-Myc and inhibits c-Myc expression

**Fig. 7:**
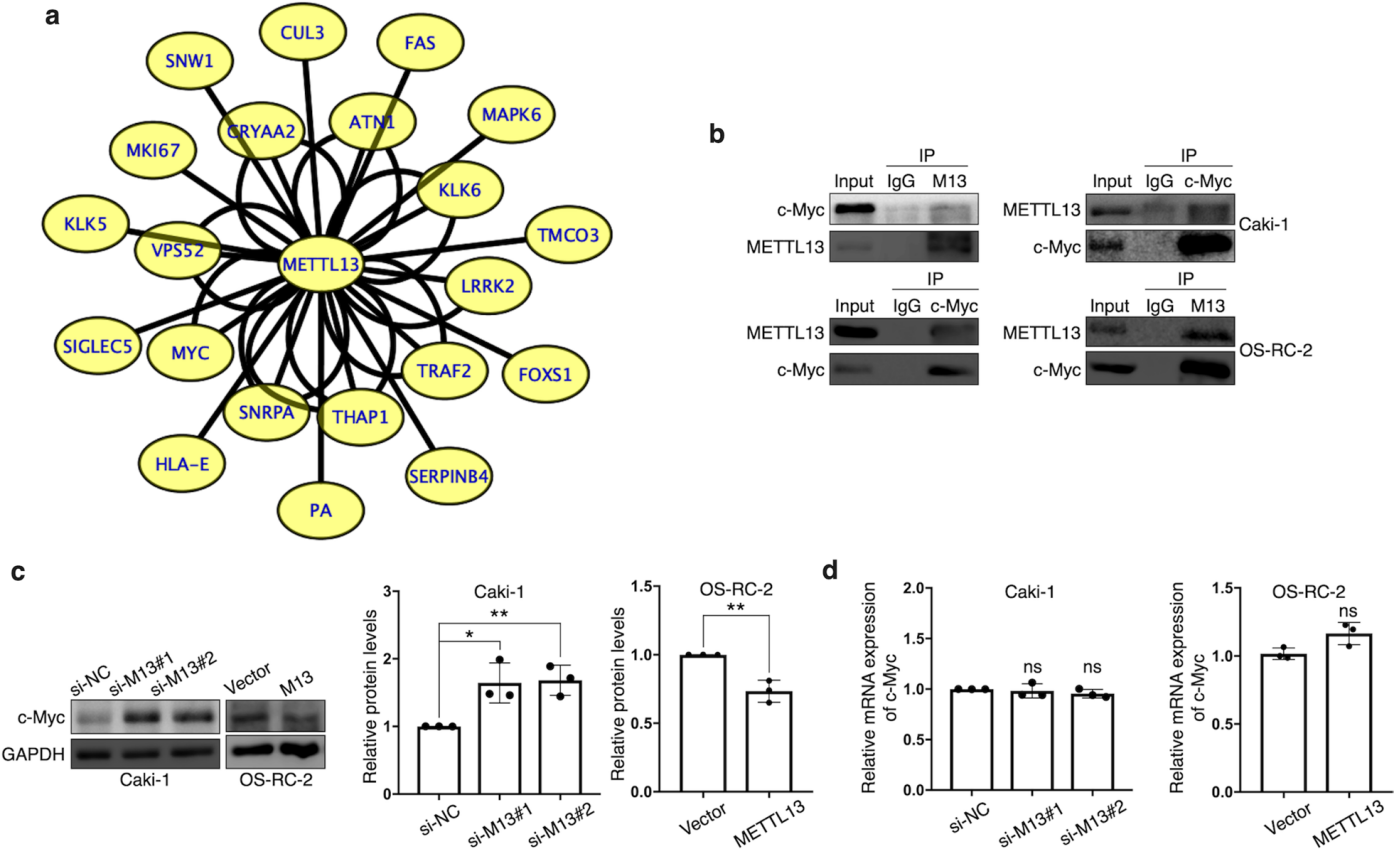
METTL13 binds with c-Myc and inhibits c-Myc expression. **a** Genes interacting with METTL13 were provided by database IntAct (https://www.ebi.ac.uk/intact/) and visualized by software Cytoscape. Each string represents an interaction accession. **b** The interaction between METTL13 and c-Myc was confirmed by co-immunoprecipitation (Co-IP). **c**, **d** Protein levels (**c**) and mRNA levels (**d**) of c-Myc were respectively detected by western blot and qRT-PCR after silencing or overexpressing METTL13. Each experiment was performed independently triplicate. Student’s t-test (two-tailed) was used for statistical analysis, *P < 0.05, **P < 0.01, ***P < 0.001. Bar graphs: mean ± S.D., n = 3

Meanwhile, METTL13 has been confirmed to have properties of protein binding and protein modification [11, 12]. Out of great interest, we surveyed IntAct [19], a protein-protein interaction database and we displayed a number of protein interactions of METTL13, which was visualized by software Cytoscape [20] and shown in Fig. 7a. According to this result, we selected c-Myc, the most classic member of Myc family, as a target interacting with METTL13 to investigate because of its critical role in tumorigenesis and metabolism. By performing co-immunoprecipitation assay, we were convinced that METTL13 could physically bind to c-Myc (Fig. 7b). Furthermore, results showed that silencing METTL13 resulted in increase in c-Myc protein levels in Caki-1 cells and on the contrary in OS-RC-2 cells, the overexpression of METTL13 led to an opposite phenomenon (Fig. 7c), which indicated an inhibitory effect that METT13 had on c-Myc protein expression. Similarly, after alterations of METTL13 expression, no significant impact on c-Myc mRNA expression levels was observed (Fig. 7d), suggesting a post-transcriptional modification of c-Myc by METTL13. In conclusion, METTL13 seems to physically interact with c-Myc and to negatively regulate c-Myc protein expression in ccRCC, while many other potential interactors of METTL13 are worthy of further studies.

### METTL13 inhibits tumor growth and metastasis in vivo

**Fig. 8:**
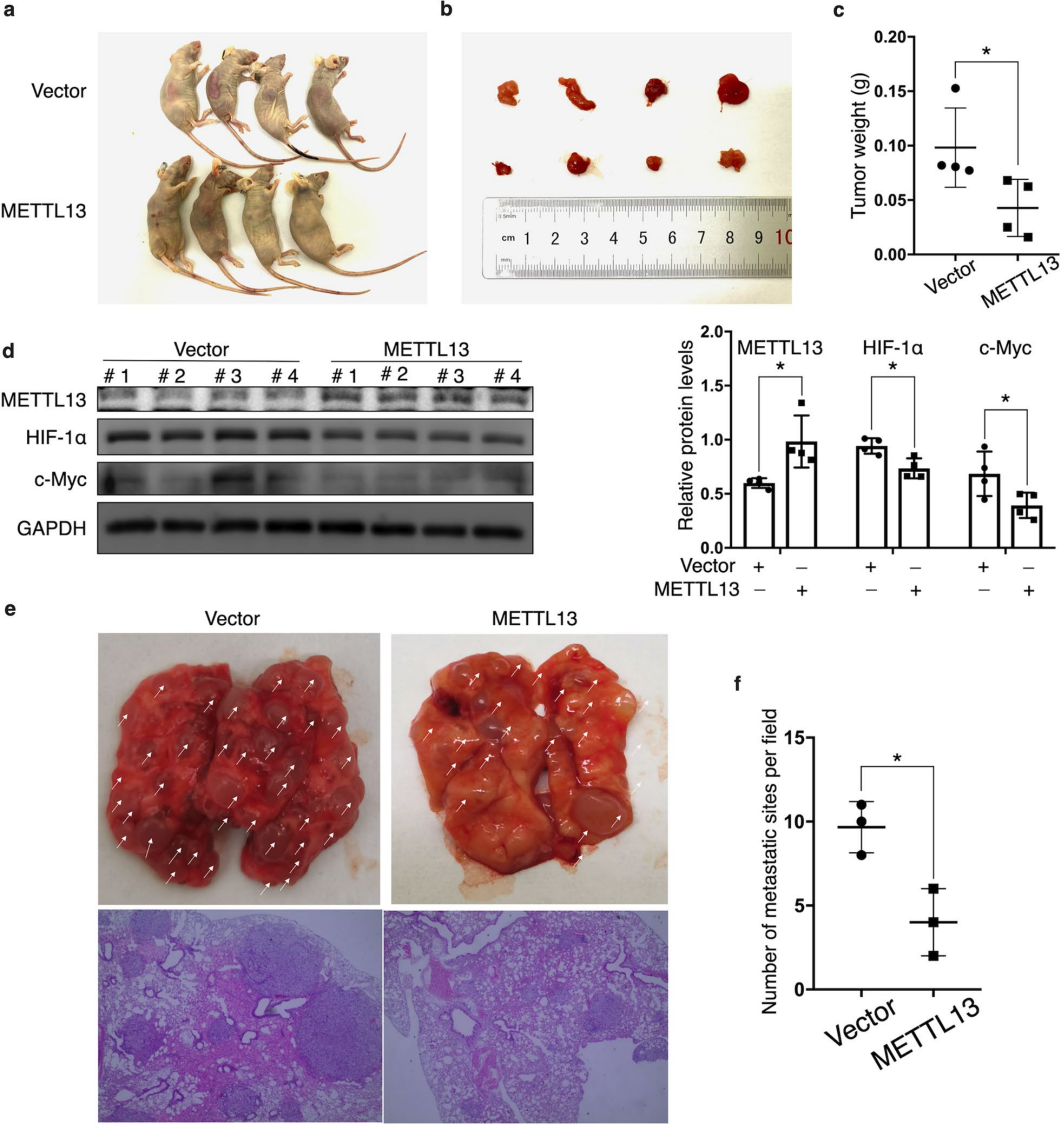
METTL13 inhibits ccRCC cells’ growth and metastasis *in vivo*. **a** Image of BALB/c nude mice executed 30 days after injection of cells transfected with empty vector or with METTL13 overexpression plasmid. **b** Image of excised tumors from the mice. **c** The weights of the isolated tumors. **d** western blot was used to measure protein expressions of METTL13, HIF-1α and c-Myc in the nude mice tumors. **e** Cells overexpressing empty vector or METTL13 were injected into nude mice through tail vein. Representative images of lungs with metastatic tumors excised from the mice and H&E staining of the lung tissues were shown. **f** The numbers of metastatic tumors in lungs per field were measured under a magnification of 40x. Each experiment was conducted at least three times. Student’s t-test (two-tailed) was used for statistical analysis, *P < 0.05. Bar graphs: mean ± S.D., n = 3

To further investigate the biological functions of METTL13 *in vivo*, OS-RC-2 cells stably overexpressing METTL13 were subcutaneously injected into BALB/c nude mice while OS-RC-2 cells transfected with empty vector was processed in the same way as the negative control. After 4 weeks, tumors from the METTL13 overexpression group showed significantly smaller sizes and lower weights by comparison to tumors from the negative control group (Fig. 8a–c). After extracting proteins from the tumors and performing western blotting assay, we detected significantly lower protein expressions of HIF-1α and c-Myc in the METTL13 overexpression group (Fig. 8d), which was accordant to what we previously observed by experiments *in vitro*. Next, teil vein injection was performed. METTL13-overexpressed and empty vector-expressed OS-RC-2 cells were respectively injected into BALB/c nude mice, 45 days after which lung colonization was analyzed. The result showed that lungs excised from mice in the METTL13 overexpression group were observed with presence of fewer metastatic tumors (Fig. 8e, f), suggesting METTL13’s inhibitory effect on metastatic ability of ccRCC cells.

## Discussion

Despite the oncogenic role played by METTL13’s enhanced expression in many types of tumors, this molecule is underexpressed in ccRCC and its low expression is associated with poor prognosis according to public datasets, which aroused our interest in further research. By using qRT-PCR and western blotting assay, we detected significant decreases in mRNA and protein levels of METTL13 in ccRCC tissues compared to normal adjacent tissues as well as a negative relevance between METTL13 expression and malignancy grades of ccRCC via immunohistochemistry. *In vitro* and *in vivo* studies confirmed the inhibitory effect that METTL13 had on ccRCC cells’ proliferation and metastasis. Furthermore, the alteration of ccRCC cells’ metastatic ability by METTL13 might result from the regulation of epithelial-mesenchymal transition (EMT). Based on these, we identify METTL13 as a tumor suppressor gene in clear cell renal cell carcinoma, affecting a variety of biological behaviors of cancer cells and contrary to its role in most other cancers. Therefore, METTL13 is likely to act as a new biomarker for ccRCC in the future.

By performing bioinformatic analyses with transcriptome data provided by TCGA datasheet, we found that METTL13 had a great potential to participate in metabolism regulation, including glycolysis, gluconeogenesis, TCA cycle, fructose and mannose metabolism, which had been proved to be closely associated with occurrence and development of ccRCC [21–24]. What appealed to us was that HIF-1 signaling pathway was involved and it’s also been reported to play an important role in cancer metabolism [25, 26]. HIF-1α, one of the most important molecules in the HIF-1 signaling pathway, has brought about scholars’ interest with its crucial roles in various diseases, including malignancies [27–30]. Previous studies have stated HIF-1α’s abnormal overexpression in ccRCC tissues and have demonstrated its tumorigenic functions as well as multiple molecular mechanisms [31–34]. According to our experiments, the expression of HIF-1α was observed to be negatively regulated by METTL13 at post-transcriptional level. Additionally, the PI3K/AKT/mTOR signaling pathway, which has been confirmed to promote HIF-1α protein translation, was observed to be inactivated by METTL13. However, it still requires further efforts to determine whether METTL13 regulates HIF-1α expression via other mechanisms or not and how METTL13 specifically participates in the whole HIF-1 signaling pathway. Besides, the roles that it plays in other biological behaviors and processes of ccRCC, especially metabolism, are left to discover. According to the WGCNA result, we also suppose that METTL13 is able to regulate cell adhesion in ccRCC by possibly connecting to other molecules. Thus, inhibition of ccRCC metastasis by METTL13 may result from other mechanisms besides the EMT alteration.

Furthermore, the database IntAct provided us with more than 20 proteins potentially combining with METTL13. Among these interactors, TRAF2 influences mitochondrial apoptosis of ccRCC [35]; KLK6’s expression is negatively associated with renal carcinoma grades [36]; higher HLA-E mRNA level predicts better prognosis of ccRCC patients [37]; FAS can be potentially regarded as a biomarker for predicting survival of renal cancer patients who have received nephrectomy [38]. The result also involved Myc, a family of proto-oncogenes, which extensively functions in cancer formation and development [39]. c-Myc is the most classic and important member of the Myc family and it has been reported to control various biological behaviors of ccRCC cells with abundant mechanisms including metabolism regulation [40–43]. Via our experiments, we evidenced not only the physical interaction between METTL13 and c-Myc but also the restraining effect that METTL13 had on c-Myc protein expression. However, the specific mechanism still remains a mystery.

Based on our findings, METTL13 is of great potential to act as a new biomarker for ccRCC diagnosis and therapy. On the one hand, the expression level of METTL13 in ccRCC is likely to be considered a potential molecular indicator in the future, which may assist pathologists in diagnosing clinicopathological characters of the tumors and predicting patients’ prognosis; on the other hand, we could propose new methods using METTL13 agonists, taking advantage of METTL13's tumor suppressing role in ccRCC for renal cancer therapy. Besides, with an increasing number of studies aimed at metabolism regulation in renal carcinoma [21–24], we are looking forward to a new therapeutic strategy for ccRCC by utilizing METTL13’s potential participation in metabolism, including METTL13’s inhibitory impact on expressions of metabolism-related genes, HIF-1α and c-Myc.

## Conclusion

Collectively, our research demonstrated METTL13’s tumor suppressing role in clear cell renal cell carcinoma for the first time, as featured by inhibiting growth and metastasis of cancer cells. In addition, we summarized a variety of biological processes and molecular mechanisms that METTL13 might be involved in, according to which we validated its inhibitory effect on PI3K/AKT/mTOR/HIF-1α pathway. Moreover, we confirmed that METTL13 could bind to c-Myc and restrain its expression. On the basis of these, METTL13 has a great potential to act as a new diagnostic biomarker and therapeutic target for ccRCC in the future, while its molecular mechanisms are worthy of further validation and investigation.

## Supplementary Information

**Additional file1**: Approval by Research Ethics Committee of China Medical University.

**Additional file2**: **Table S1**. Sequences of qRT-PCR primers.

**Additional file3**: **Table S2**. Information of primary antibodies.

## Data Availability

The datasets used and/or analyzed during the current study are available from the corresponding author on reasonable request.

## Abbreviations

ccRCC: Clear cell renal cell carcinoma
METTL13: Methyltransferase like 13
HIF-1α: Hypoxia-inducible factor-1α
c-Myc: Myelocytomatosis virus oncogene cellular homolog
5-EdU: Ethynyl-2′-deoxyuridine
qRT-PCR: Quantitative real-time PCR
IHC: Immunohistochemistry
WGCNA: WEIGHTED gene co-expression network analysis
Co-IP: Co-immunoprecipitation
PI3K: Phosphatidyl inositol-4,5-bisphosphate-3-kinase
mTOR: Mammalian target of rapamycin
AKT: Protein kinase B
