# Supplementary figures and images for: METTL13 inhibits progression of clear cell renal cell carcinoma with repression on PI3K/AKT/mTOR/HIF-1α pathway and c-Myc expression
Source: J Transl Med. 2021 May 13;19:209. doi: 10.1186/s12967-021-02879-2 (PMC8120818; doi:10.1186/s12967-021-02879-2)

Supplementary file 1. Approval by Research Ethics Committee of China Medical University.


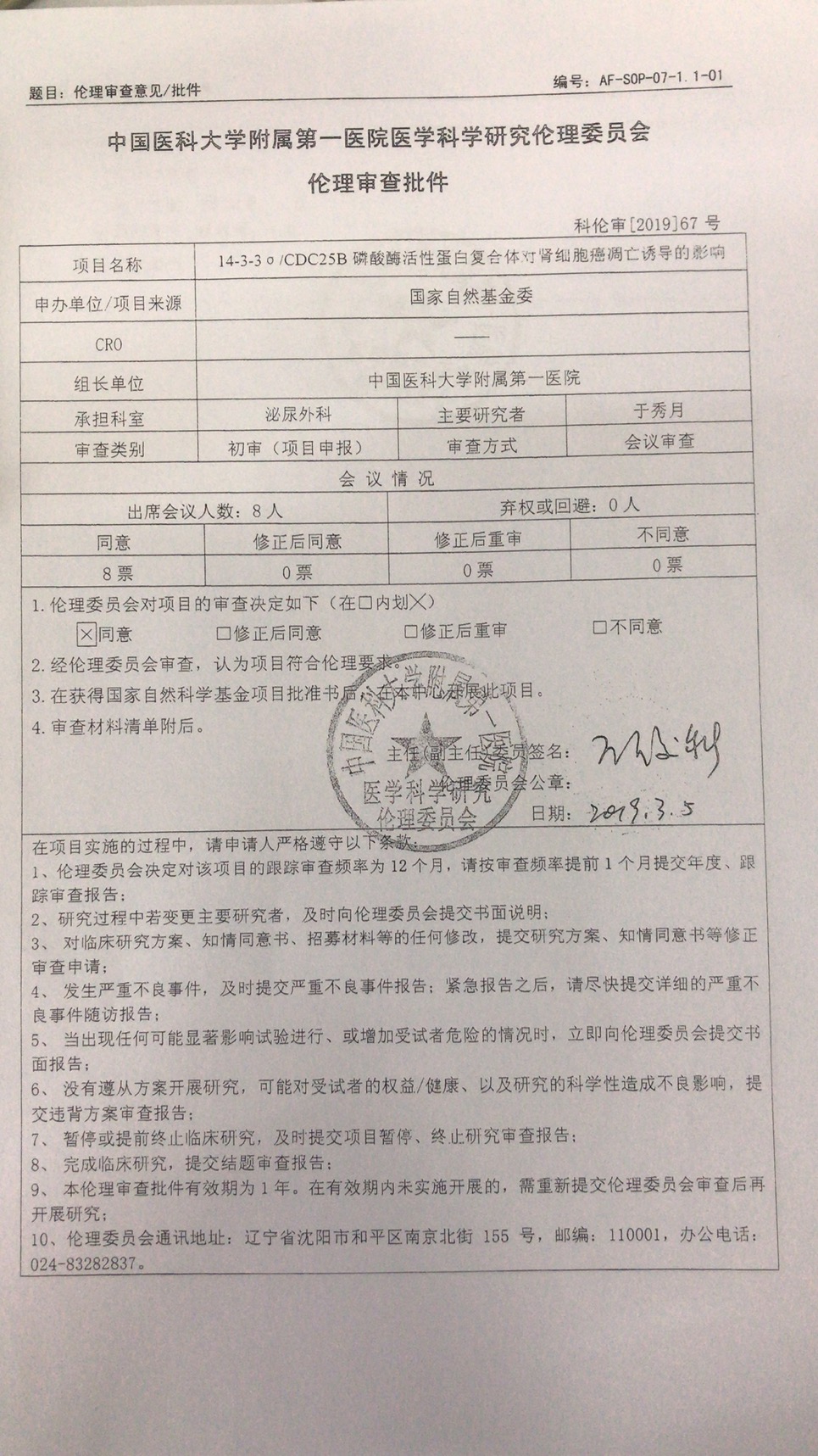

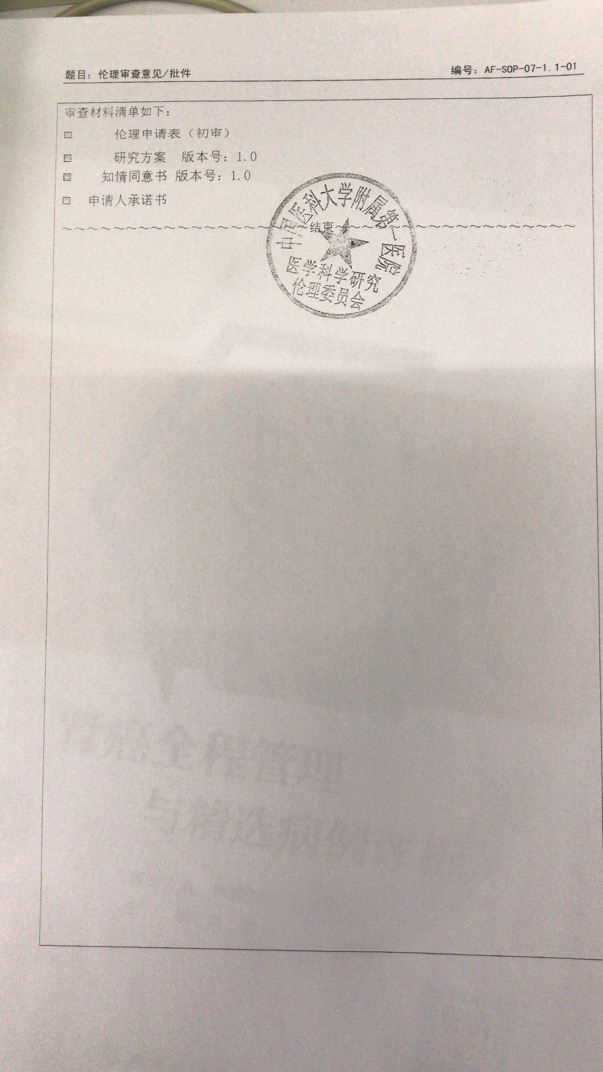

Supplement: Supplementary file 1 — Additional file1: Approval by Research Ethics Committee of China Medical University. [file 12967_2021_2879_MOESM1_ESM.docx]
