# Supplementary material for: METTL13 inhibits progression of clear cell renal cell carcinoma with repression on PI3K/AKT/mTOR/HIF-1α pathway and c-Myc expression
Source: J Transl Med. 2021 May 13;19:209. doi: 10.1186/s12967-021-02879-2 (PMC8120818; doi:10.1186/s12967-021-02879-2)
Supplement: Supplementary file 2 — Additional file2: Table S1. Sequences of qRT-PCR primers. [file 12967_2021_2879_MOESM2_ESM.docx]

**Supplementary Table 1. Sequences of qRT-PCR primers.**

| Primers | Forward sequences | Reverse sequences |
| --- | --- | --- |
| METTL13 | CAGGAGGTTGATTACAGTGGC | CTCCATGACTCTAGCCGACA |
| HIF-1α | GCACAGGCCACATTCACG | TTCACAAATCAGCACCAAGC |
| Myc | GGCTCCTGGCAAAAGGTCA | CTGCGTAGTTGTGCTGATGT |
| β‐actin | CATGTACGTTGCTATCCAGGC | CTCCTTAATGTCACGCACGAT |
| GAPDH | ACAACTTTGGTATCGTGGAAGG | GCCATCACGCCACAGTTTC |
