# Supplementary material for: METTL13 inhibits progression of clear cell renal cell carcinoma with repression on PI3K/AKT/mTOR/HIF-1α pathway and c-Myc expression
Source: J Transl Med. 2021 May 13;19:209. doi: 10.1186/s12967-021-02879-2 (PMC8120818; doi:10.1186/s12967-021-02879-2)
Supplement: Supplementary file 3 — Additional file3: Table S2. Information of primary antibodies. [file 12967_2021_2879_MOESM3_ESM.docx]

**Supplementary Table 2. Information of primary antibodies.**

| Antibodies | Source | Identifier |
| --- | --- | --- |
| METTL13 | GeneTex | Cat# GTX120626; RRID: AB_10732544 |
| β‐actin | Proteintech | Cat# 66009-1-lg; RRID: AB_2687938 |
| GAPDH | Cell Signaling Technology | Cat# 5174S; RRID: AB_10622025 |
| N-cadherin | Abcam | Cat# ab76011; RRID: AB_1310479 |
| E-cadherin | Abcam | Cat# ab40772; RRID: AB_731493 |
| HIF-1α | Santa Cruz Biotechnology | Cat# sc-71247; RRID: AB_1124904 |
| c-Myc | Santa Cruz Biotechnology | Cat# sc-40; RRID: AB_627268 |
| p-PI3 Kinase p85 | Cell Signaling Technology | Cat# 4228S; RRID: AB_659940 |
| PI3 Kinase p85 | Cell Signaling Technology | Cat# 4257; RRID: AB_659889 |
| p-AKT1/2/3 | Santa Cruz Biotechnology | Cat# sc-7985; RRID: AB_667741 |
| Akt1/2/3 | Santa Cruz Biotechnology | Cat# sc-8312; RRID: AB_671714 |
| p-mTOR (Ser2448) | Cell Signaling Technology | Cat# 5536S; RRID: AB_10691552 |
| mTOR | Cell Signaling Technology | Cat# 2983; RRID: AB_2105622 |
